# Medical Fees—No. 2

**Published:** 1845-11-01

**Authors:** 


*Medical Fees—No.* 2.—The importance of this topic grows upon us with farther reflection. By a singular coincidence, on perusing the Boston Journal, received on the day on which our last was issued, we found an editorial article on the subject, embodying several of the same ideas which we had simultaneously presented; and in a later number we observe a communication from a correspondent who signs himself *Senex*, giving some wholesome advice to the younger portion of the profession. The truth is, the medical profession would be elevated, and rendered more useful, if a reform could be effected in the system of remuneration. If the mass of its members were better paid for the amount of labor which they perform, and they were relieved of the additional drudgery and anxiety imposed upon them by the present system of interminable credit, the profession would hold a higher position in public estimation, medical services would be better appreciated, and, in fact, possess more value than now belongs to them. The criterion by which most persons judge of the worth of any thing is a certain pecuniary valuation. Medical services can be hardly said to have any such valuation, the rule being to charge a certain amount, and collect such a proportion of the amount charged as may comport with the patient's own estimation of the services, or with his disposition to pay. As is justly remarked by Senex in the article referred to in the Boston Journal, the profession itself, is, to a considerable extent, at fault in this matter. Physicians have been too ready to adapt themselves to the derogatory views which a considerable portion of the community entertains on this subject; and, perhaps, to extend their practice, they sometimes contribute directly to promote these views, by depreciating the value of their own services. The latter course is as impolitic as it is dishonorable, since, in the end, it is sure to recoil upon those who adopt it.

The reformation required, is, simply, this: let it be understood with the profession, and the public, that medical services have a certain specific value, and that a physician's demand is entitled to settlement so soon as the service is rendered. Let it be considered an invariable duty on the part of the patient to call upon the physician directly he ceases his attendance, and either liquidate the debt which has accrued, or arrange for its payment. In all cases where advice is given, or a single visit made, the fee should be paid at the time. It is not, of course, to be expected that all patients, at all times, will be in a situation to discharge medical dues with this promptness; and it will follow, also, as a matter of course, that the amount charged will have to be varied in certain cases to meet the different circumstances of different individuals. But let the principle be established that the physician expects his remuneration immediately, and, if it be not convenient to conform to this principle, it belongs to the patient to make the advances, and without delay, toward a mutual understanding and settlement according to circumstances.

Will the public oppose such a reformation? They will not who feel a proper sense of gratitude for the attentions which they have received, and are willing to reward these attentions according to their ability. They, alone, who experience no such sentiments, and have no disposition to pay if they can avoid it, will find fault with an alteration such as is proposed. The feelings and views of this class of persons are of little account in the consideration of the subject. It is precisely this class of persons that we wish to reach by a reform, and we cannot expect to compass them with their own consent. But we can reach them, *nolens volens*, by adopting the no-credit system, and uniting in refusing our services to them on any other terms. There is no reason to anticipate any opposition to the change from those whose cooperation and respect are in any way desirable. All that we have to do, then, is to arrange the matter among ourselves, publish our intentions, and hold each other to a strict performance of our mutual engagements. This can readily be done through the associations already existing. We deem the subject sufficiently important in itself and its relations, to come before the contemplated National Convention. We should be glad to have this Convention take it into consideration, recommend suitable measures, and then, through the various local associations, the reform could be made universal and effectual.

